# Alterations of the MEK/ERK, BMP, and Wnt/β-catenin pathways detected in the blood of individuals with lymphatic malformations

**DOI:** 10.1371/journal.pone.0213872

**Published:** 2019-04-04

**Authors:** Taehan Kim, Elidia Tafoya, Malcolm P. Chelliah, Ramrada Lekwuttikarn, Jiang Li, Kavita Y. Sarin, Joyce Teng

**Affiliations:** 1 Department of Dermatology, Stanford University School of Medicine, Stanford, California, United States of America; 2 Stanford University School of Medicine, Stanford, California, United States of America; University of PECS Medical School, HUNGARY

## Abstract

Lymphatic malformation (LM) is a developmental anomaly of the lymphatic system that may lead to disfigurement, organ dysfunction and recurrent infection. Though several treatment modalities exist, pharmacotherapy is often associated with side effects and recurrence is common following surgical interventions. Moreover, despite the recent discovery of *PIK3CA* mutations in lymphatic endothelial cells of LM patients, the full spectrum of molecular pathways involved in LM pathogenesis is poorly understood. Here, we performed RNA sequencing on blood samples obtained from ten LM patients and nine healthy subjects and found 421 differentially expressed genes that stratify LM subjects from healthy controls. Using this LM gene signature, we identified novel pathway alterations in LM, such as oxidative phosphorylation, MEK/ERK, bone morphogenetic protein (BMP), and Wnt/β-catenin pathways, in addition to confirming the known alterations in cell cycle and the PI3K/AKT pathway. Furthermore, we performed computational drug repositioning analysis to predict existing therapies (e.g. sirolimus) and novel classes of drugs for LM. These findings deepen our understanding of LM pathogenesis and may facilitate non-invasive diagnosis, pathway analysis and therapeutic development.

## Introduction

Vascular anomalies represent a heterogeneous group of disorders that can be classified broadly into vascular tumors and vascular malformations [1]. Vascular malformations are subdivided into arterial, capillary, venous, lymphatic and combined malformations according to the type of vessel affected [2]. Lymphatic malformations (LMs) are dilated lymphatic cystic channels that are filled with lymph but are not appropriately connected to the circulatory network [3, 4]. As a consequence, these lesions lead to fluid accumulation and edema, often resulting in pain and infection. LMs are believed to arise from abnormal development of the lymphatic system and account for approximately 2.8/100,000 hospital admissions [5, 6]. LMs commonly present in locations with increased lymphatic drainage such as head and neck, axilla, groin, mediastinum and retroperitoneum [7]. There are three subtypes of LMs: macrocystic, microcystic and combined. Macrocystic LMs are large (> 1 cm), smooth, translucent, multilobular lesions while microcystic LMs contain clusters of smaller cystic structures that permeate the subcutaneous tissue and muscles [7, 8]. Combined LMs contain a mixture of micro- and macrocystic lesions and are often seen below the head and neck region. LMs can present either as an isolated vascular anomaly or as part of overgrowth syndromes that arise due to mutations in the PI3K/AKT pathway, such as Proteus syndrome, Klippel-Trenaunay syndrome (KTS) and congenital lipomatous overgrowth with vascular, epidermal, and skeletal anomalies (CLOVES) syndrome [4].

Researchers have recently identified activating mutations in *PIK3CA* in isolated LMs as well as overgrowth syndromes such as KTS and CLOVES syndrome, and collectively termed these diseases *PIK3CA*-related overgrowth spectrum (PROS) [9]. According to two independent studies, *PIK3CA* mutations are present in roughly 95% of LMs, with five activating hotspot mutations accounting for a majority of the cases [10, 11]. Despite the ubiquitous nature of these mutations in LM, the full spectrum of molecular pathway alterations is not well characterized.

Though the majority of LMs follow a benign clinical course, severe LMs may cause complications such as disfigurement, organ dysfunction and recurrent infection, leading to life-threatening conditions. Current treatment modalities include excision, pharmacotherapy, and sclerotherapy, and treatment options depend on the size and type of lesion, anatomic location and risk of complications. Complete excision of the lesion is often difficult, and recurrence rates are as high as 40% for incomplete excision and 17% for complete excision [12]. A number of medications have been used to treat LMs with mixed results, including propranolol, sildenafil and sirolimus (rapamycin) [1, 13, 14]. In particular, sirolimus, which directly targets mTOR of the PI3K/AKT/mTOR axis, has gained interest as an effective therapy for complicated LMs. In a recent prospective phase II clinical trial that examined the efficacy and safety of 11-month course of sirolimus, researchers observed partial response in 7/7 and 2/5 patients with generalized lymphatic anomaly and microcystic lymphatic malformation, respectively [15]. Sirolimus was also relatively well tolerated, causing no toxicity-related deaths 5 years after treatment [15].

LMs are often associated with risk of edema and cellulitis following upper respiratory viral infection, suggesting a propensity for a systemic inflammatory response. Therefore, we sought to determine if there is a LM-specific gene signature detectable in peripheral blood cells by performing RNA-sequencing (RNA-Seq) on blood samples of LM patients and healthy subjects. Here we demonstrate an LM gene signature in the blood that can stratify individuals with LM from healthy subjects. Consistent with previous studies, the PI3K/AKT pathway and cell cycle regulation are among the pathways involved in LMs. However, we also identify novel pathways including the MEK/ERK, bone morphogenetic protein (BMP), Wnt/β-catenin, and ephrin signaling pathways involved in LM formation. Finally, we employ this signature to predict potential therapeutics for LM and confirm existing therapies, which further validates our results.

## Materials and methods

### Patient recruitment

Ten LM patients (age 2–22 years old) seen at the vascular anomaly clinic at Stanford Hospital were enrolled in this study. None of the participants had surgery or medical treatment at least 6 months prior to the time of enrollment. Following approval by the Stanford Human Subjects IRB, the participants or their legal guardians in this manuscript have given written informed consent (as outlined in PLOS consent form) to publish these case details. One of the patients (LM 7) provided written consent to be enrolled in the study but declined to have her photograph published.

### Sample processing and RNA-Seq

Whole blood was collected in Tempus RNA tubes and stored at -80°C until RNA extraction, which was performed for all samples simultaneously by the Stanford Functional Genomics Facility (SFGF). RNA quantification and quality assurance were performed on an Agilent 2100 Bioanalyzer, and library construction was completed using the Illumina preparation kit according to the manufacturer’s instructions. Libraries were PolyA-enriched and followed by depletion of globin transcripts and ribosomal RNA. Paired-end sequencing of 75 base pair fragments was performed on the Illumina Nextseq, with an average coverage of 53 million reads per sample. Reads were aligned to the human genome (hg19) using Tophat (v2.1.1) [16] with an average of 89.6% of the reads mapping. Featurecounts (v1.5.2) [17] was used to generate count data, and DESeq2 (v1.6.3) [18] was used to identify differentially expressed genes between the LM and normal blood samples.

### Hierarchical clustering and pathway analysis

A total of 421 genes (253 upregulated and 168 downregulated) were selected using a threshold of Benjamini-Hochberg adjusted p-value ≤ 0.05. FPKM (fragments per kilobase of transcript per million mapped reads) of these genes were generated from Cufflinks (v 2.2.1) [19] and inputted into GENE-E (Broad Institute, Cambridge, MA; https://software.broadinstitute.org/GENE-E/index.html, last access on 2/2/2019), and hierarchical clustering analysis was performed using one minus Pearson correlation [20] and city-block distance [21] for row and column clustering, respectively. Gene clusters were analyzed using Ingenuity Pathway Analysis (IPA^®^ version 01–12, QIAGEN Redwood City) to identify pathways associated with each cluster. Upregulated genes in LM were converted to their corresponding probe set IDs (Affymetrix Human Genome U133 Plus 2.0) and analyzed using DAVID Functional Annotation Tool (http://david.ncifcrf.gov, last access on 9/21/2018) [22, 23] to identify Kyoto Encyclopedia of Genes and Genomes (KEGG) pathways associated with the genes [24]. Upstream regulators were determined using IPA expression analysis.

### Immunohistochemistry

Histology was performed by HistoWiz Inc. (histowiz.com) using standard operating procedures and fully automated workflow. Samples were processed, embedded in paraffin, and sectioned at 4 μm. Immunohistochemistry was performed on a Bond Rx autostainer (Leica Biosystems) with enzyme treatment (1:1000) using standard protocols. Antibodies used were rabbit anti-p-4E-BP1 (Thr37/46) (Cell Signaling, #2855, 1:800), rabbit anti-p-ERK1/2 (Thr202/Tyr204) (Cell Signaling, #4370, 1:100), and rabbit anti-β-catenin (Cell Signaling #8480, 1:100) antibodies. Bond Polymer Refine anti-rabbit HRP Detection (Leica Biosystems) was used according to manufacturer’s protocol. Sections were then counterstained with hematoxylin, dehydrated and film coverslipped using a TissueTek-Prisma and Coverslipper (Sakura). Whole slide scanning (40x) was performed on an Aperio AT2 (Leica Biosystems). The images were quantified using Halo image analysis software (Indica Labs) using CytoNuclear module.

### Computational drug repositioning analysis

Genes that meet the criteria (adj. p-value ≤ 0.05; Log_2_FoldChange < -0.33 or > 0.33) were entered into the Query tool on CLUE (Connectivity map Linked User Environment; https://clue.io/, last access on 9/21/2018) [25], and 744 FDA-approved drugs were selected from 2513 unique pharmacological perturbagens. In case of multiple incidences of the same drug, the incidence with the most negative score was selected.

Chemical perturbagen signatures (all cell lines and concentrations) were obtained from iLINCS (http://www.ilincs.org/ilincs/, last access on 9/21/2018) and pooled for each drug. LM signature genes with p-values < 0.05 were selected from the pooled data, and the average Log(DiffExp) was calculated for each gene. For genes where there was no signature with p-value < 0.05, three signatures with the lowest p-values were chosen and their average Log(DiffExp) was used. Twenty genes that are opposed by either sirolimus (N = 16) or both vincristine and prednisolone (N = 4) were analyzed using IPA to identify upstream regulators.

## Results

### Clinical characteristics of LM patients

**Fig 1:**
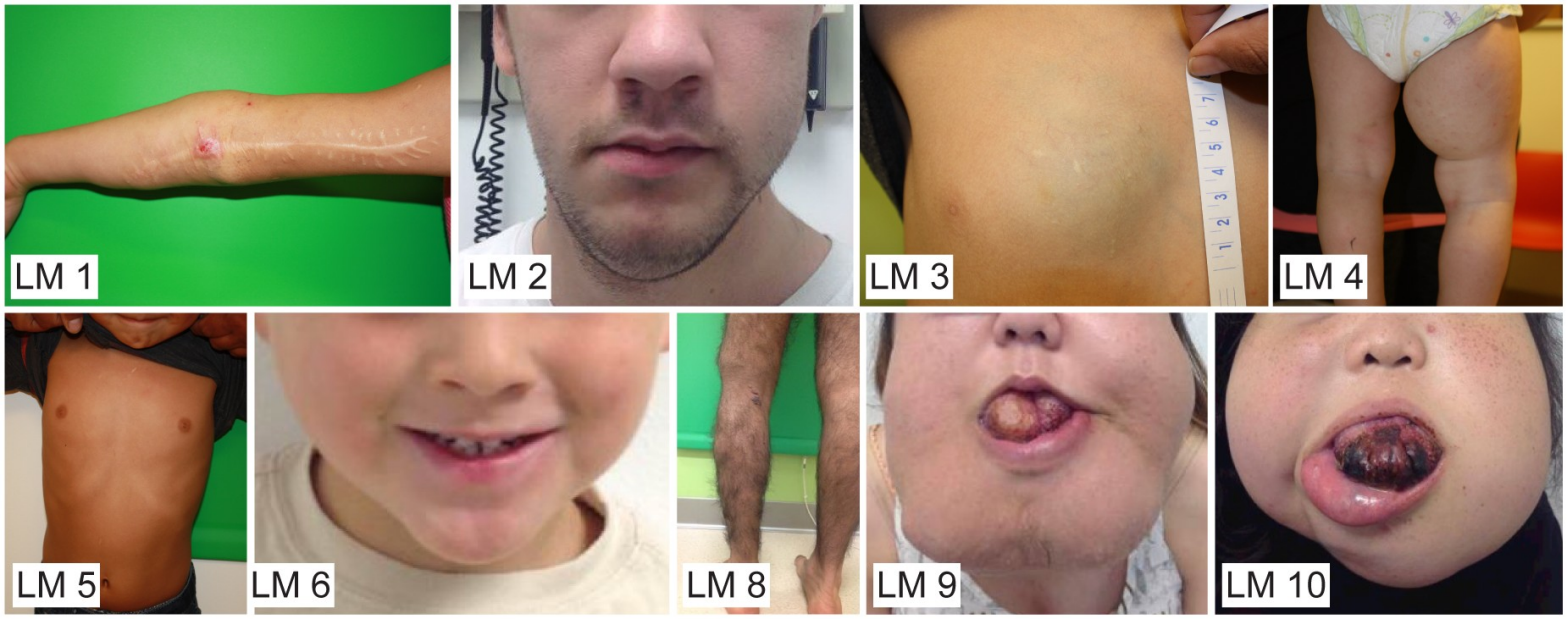
Clinical photographs of lymphatic malformation (LM) patients in this study. All patients consented to publication.

Photographs of patients are shown in Fig 1, and their demographic information and clinical characteristics are listed in Table 1. According to the ISSVA (International Society for the Study of Vascular Anomalies) classification [26], all of the patients have simple, non-combined, non-syndromic LMs without visceral involvement. Prior surgical history and exposure to pharmaceutical agents are also shown in Table 1.

**Table 1:** Clinical characteristics of lymphatic malformation (LM) patients.

	LM 1	LM 2	LM 3	LM 4	LM 5	LM 6	LM 7	LM 8	LM 9*	LM 10*
Age (y)	7	17	10	2	3	7	7	15	20	16
Sex	F	M	M	M	M	M	F	M	F	F
Weight (kg)	24	75	37	12	18	28	26	50	49	51
Overall severity^†^	Moderate	Severe	Mild	Mild	Mild	Mild	Moderate	Moderate	Severe	Severe
Type	Micro	Micro	Macro	Micro	Mixed	Micro	Micro	Micro	Micro	Micro
Location	L upper chest, arm, wrist	L chest, neck, jaw	Chest	R thigh, hip, buttock	Retro-peritoneal	R cheek	R axilla, chest	L leg, pelvis w/ OG	Bil infra-orbital, neck, chest	Bil infra-orbital. neck, chest
Size (cm^3^)	331	289	64	339	34	42	403	7,046	3,690	2,500
Depth	Infiltrative	Infiltrative	SQ	Infiltrative	Infiltrative	SQ	Infiltrative	Infiltrative	Infiltrative	Infiltrative
Venous component	-	+	-	-	-	-	-	+	-	-
Pain	+	+	-	-	-	-	-	+	+	+
Bleeding	-	-	-	-	-	-	-	-	+	+
Infection	-	+	-	-	-	+	-	-	+	+
Coagulopathy	-	-	-	-	-	-	-	+	+	+
Other Complications	Hemorrh-agic cyst	Compro-mised airway	-	Swelling, gait changes	-	-	H/o serous cystade-noma, Brenner tumor	L leg longer, thinner than R	Bone infil, trach, G-tube, L vision loss	Trach
Prior medication	SLD	SLD, SRL	SLD	-	-	SLD	SLD	-	-	SLD
Prior surgery	EX	EX, R	SCL	-	-	SCL	EX, SCL	-	EX, R, SCL	EX, R

Abbreviations: y, years; F, female; M, male; L, left; R, right; OG, overgrowth; Bil, bilateral; SQ, subcutaneous; H/o, history of; infil, infiltration; trach, tracheostomy; G-tube, gastronomy tube; SLD, sildenafil; SRL, sirolimus; EX, excision; R, surgical revision; SCL, sclerotherapy.

*Patients have very large lesions and airway involvement resulting in tracheostomy since birth and frequent infections.

^†^Overall severity: mild, LM is less than 100 cm^3^ and not associated with clinical complications; Moderate, occasional swelling and pain; severe, very large LM associated with frequent pain and cellulitis.

Coagulopathy: abnormal clotting via clinical exam, radiologic imaging, or laboratory studies (elevated D-dimer, lower fibrinogen levels).

### RNA-Seq and LM gene signature

**Fig 2:**
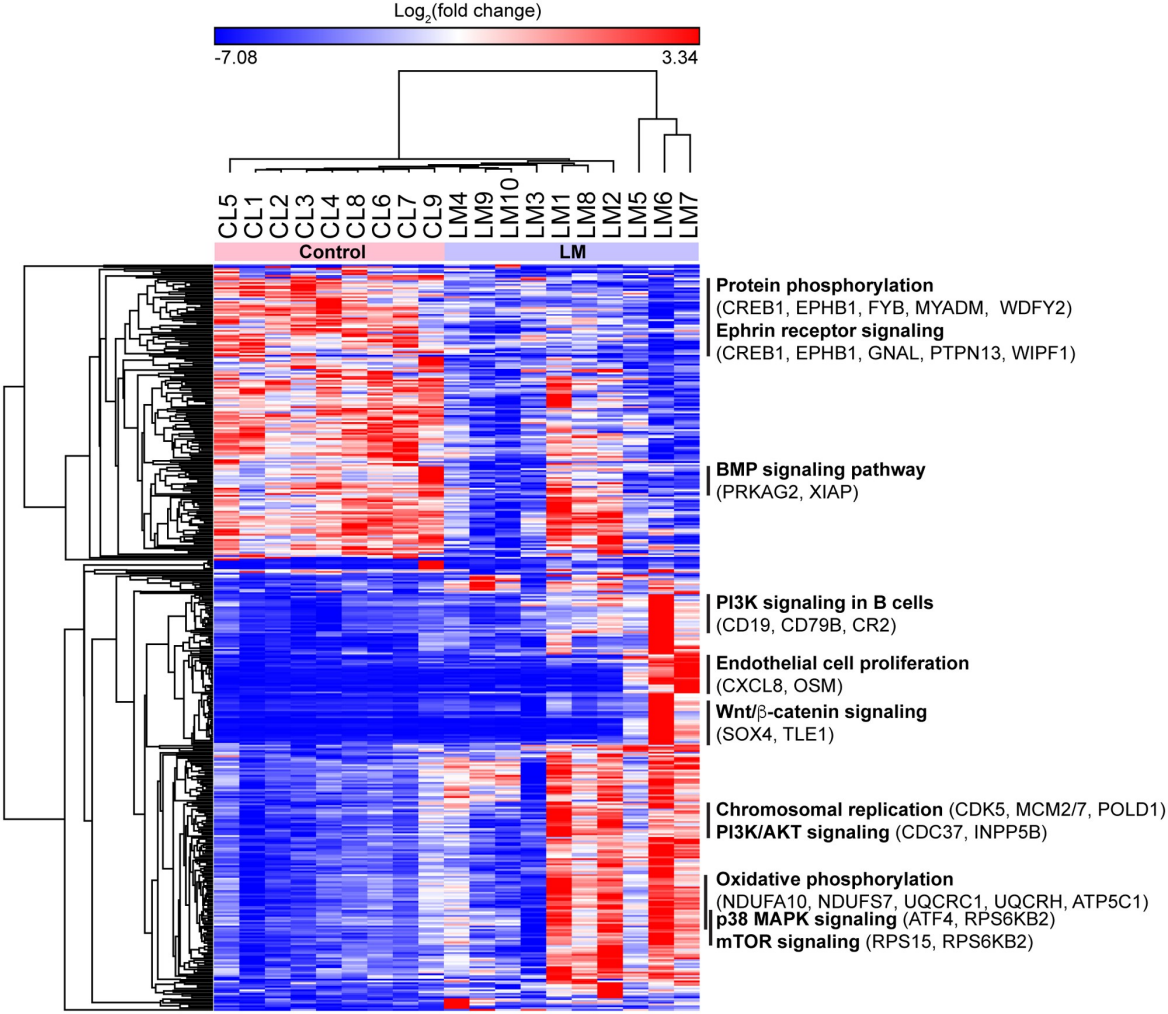
Hierarchical clustering of differentially expressed genes in blood samples of lymphatic malformation (LM) patients. A total of 421 genes were selected from RNA-Seq data using a threshold of Benjamini-Hochberg adjusted p-value ≤ 0.05. Columns and rows were clustered using city-block distance and one minus Pearson correlation as the distance metric, respectively. Canonical pathways and associated genes for each cluster were obtained using Ingenuity Pathway Analysis (IPA). Select pathways are reported including those identified in KEGG pathway analysis (Fig 3A). CL, control.

We obtained expression data for 22,722 genes by performing RNA-Seq on blood samples of 19 subjects (ten LM patients and nine healthy individuals) and identified 253 upregulated and 168 downregulated genes in LM (see S1 Table for a complete list of the genes). Unsupervised hierarchical cluster analysis on these 421 serum signature genes accurately differentiated the blood samples of LM patients from those of healthy individuals (Fig 2).

### Pathway analysis and identification of upstream regulators

**Fig 3:**
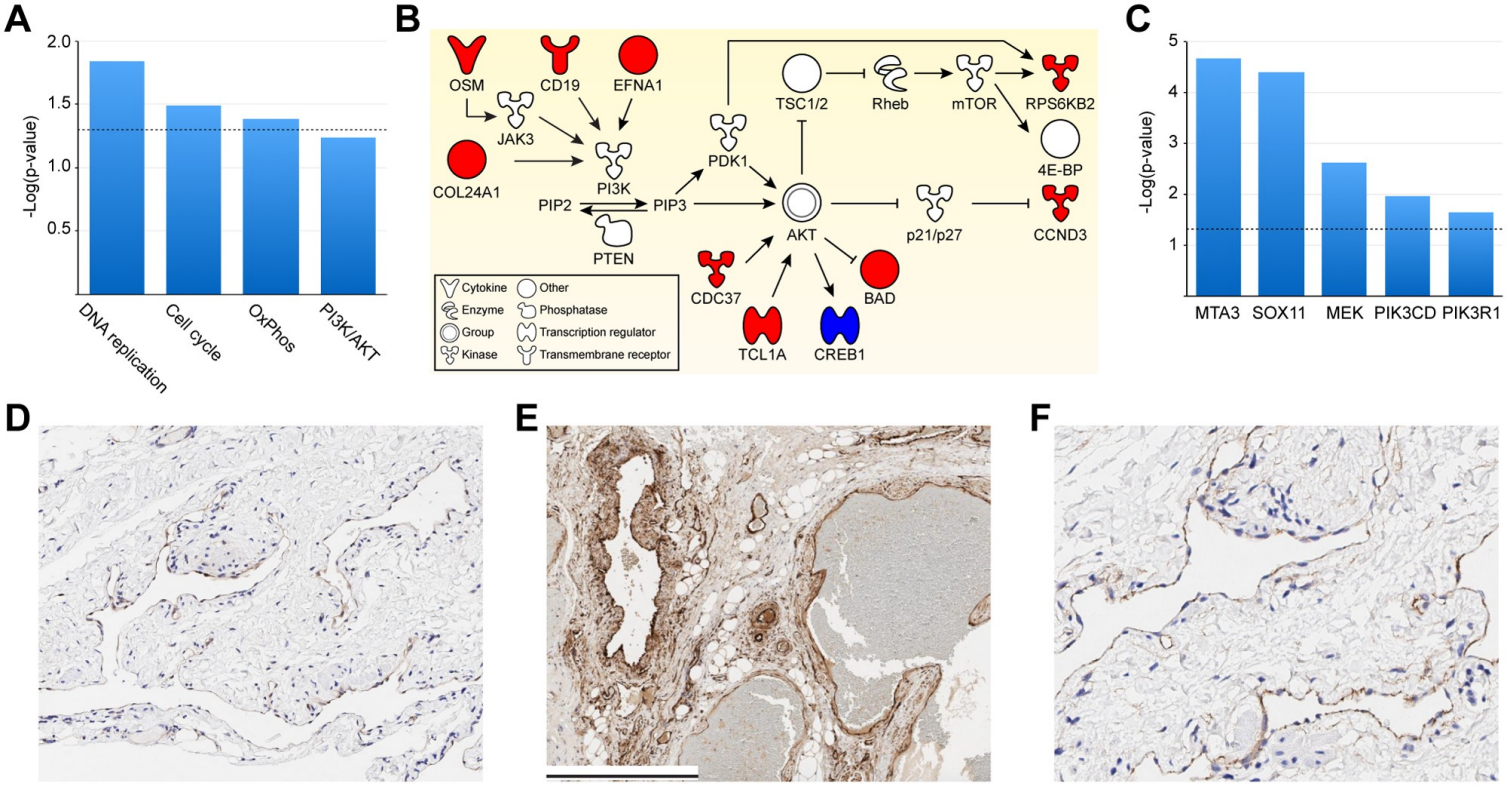
Lymphatic malformation (LM) gene signature is associated with upregulation of DNA replication, cell cycle, oxidative phosphorylation, PI3K/AKT and MEK/ERK pathways. (A) Upregulated KEGG pathways in LM and their corresponding unadjusted p-values. (B) Ten genes in the PI3K/AKT pathway are differentially expressed in LM: red, upregulated; blue, downregulated; white, no significant changes. Figure adapted from DAVID Bioinformatics Resources (https://david.ncifcrf.gov/). (C) Upstream regulators associated with the LM gene signature and their corresponding p-values of overlap. For (A), (C), dotted line indicates p-value = 0.05. (D)-(F): Immunohistochemistry of lymphatic endothelial cells shows higher levels of phospho-4E-BP1 (D) and phospho-ERK (E), and nuclear accumulation of β-catenin (F) in LM patients compared to normal skin (S2 Fig). Scale bar = 500 μm. KEGG, Kyoto Encyclopedia of Genes and Genomes. OxPhos, oxidative phosphorylation.

We then conducted pathway analysis on the LM gene signature to determine pathways that are differentially regulated between LM patients and controls. Pathways upregulated in LMs include DNA replication, cell cycle, oxidative phosphorylation and the PI3K/AKT pathway (Fig 3A). Within the PI3K/AKT/mTOR pathway, we identified ten genes that are differentially expressed in the blood samples of LM patients, including ribosomal protein S6 kinase B2 (*RPS6KB2*) and cyclin D3 (*CCND3)* (Fig 3B). In addition to the PI3K pathway, we also observed molecular alterations in the BMP, Wnt/β-catenin, and ephrin receptor signaling pathways (Fig 2). Further analysis revealed that MEK, PIK3CD, PIK3R1, MTA3, and SOX11 are among the upstream regulators associated with the 421 signature genes (Fig 3C). The downstream molecules of these regulators altered in LM are shown in S1 Fig.

To confirm the role of the PI3K/AKT/mTOR, Wnt/β-catenin, and MEK/ERK pathways in LM, we performed immunohistochemistry analysis. We observed higher expression of phospho-4E-BP1 and phospho-ERK in lymphatic endothelial cells (LECs) of LM patients compared to healthy subjects (Fig 3D and 3E; S2 Fig). We also observed nuclear localization of β-catenin in LECs of LM patients (Fig 3F).

### Computational drug repositioning analysis

We next performed computational drug repositioning analysis to identify potential therapeutics that antagonize the molecular pathways altered in LM. CLUE is a publicly available, cloud-based database that contains gene expression signatures of multiple cell lines in response to genetic or pharmacological perturbations (collectively termed perturbagens) [25]. This software algorithmically assigns a score for each perturbagen based on the similarity or dissimilarity between the perturbagen gene signature and disease signature in query. The hypothesis behind computational drug repositioning is that drugs with highly negative CLUE scores may have therapeutic potential for the disease due to their opposite gene signatures.

**Fig 4:**
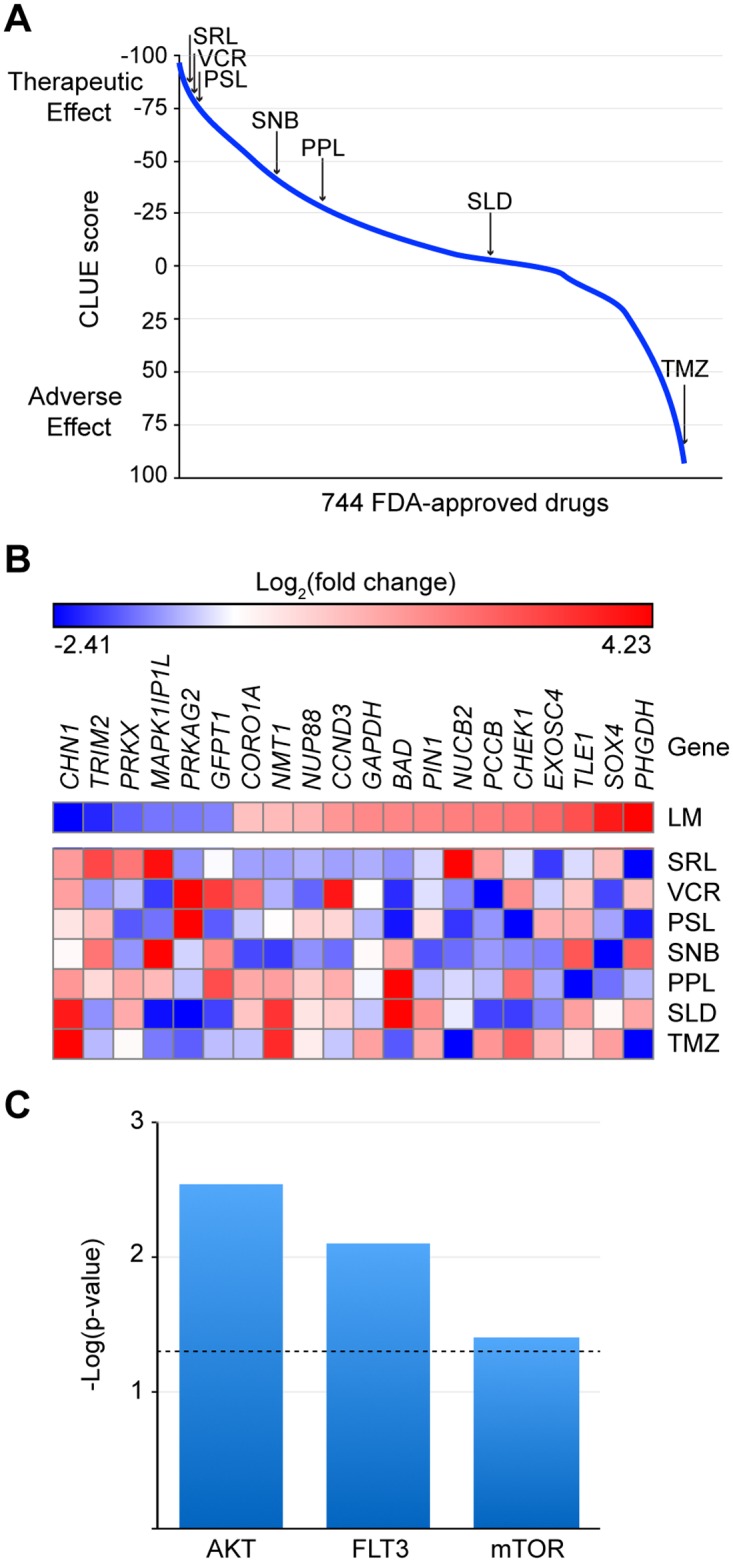
Computational drug repositioning analysis based on the lymphatic malformation (LM) gene signature predicts LM drugs and identifies AKT/mTOR inhibition as a potential mechanism of action. (A) Summary CLUE scores of drugs: SRL, -84.66; VCR, -82.70; PSL, -78.24; SNB, -40.60; PPL, -27.98; SLD, 3.10; TMZ, 91.31. TMZ is shown as an example of a drug with a similar perturbagen signature to the LM gene signature. (B), (C) Heat map (B) and upstream regulators (C) of 20 genes opposed by LM drugs. Dotted line indicates p-value of overlap = 0.05. SRL, sirolimus; VCR, vincristine; PSL, prednisolone; SNB, sunitinib; PPL, propranolol; SLD, sildenafil; TMZ, temozolomide.

We noted that most of the drugs that have shown efficacy in LM have negative CLUE scores (Fig 4A). Sirolimus has the most negative CLUE score of -84.66, followed by prednisolone, sunitinib, propranolol and sildenafil. Interestingly, vincristine, a chemotherapy drug that has been used to treat refractory vascular tumors, shows a CLUE score comparable to that of sirolimus. Other FDA-approved drugs that are predicted to antagonize molecular pathways altered in LM include JAK inhibitors, calcium channel blockers, and KATP activators, suggesting alternative approaches to consider for LM treatment (S2 Table). Among pre-clinical drugs, we found that BMP and Wnt modulators are among the drugs with the most negative CLUE scores. Dorsomorphin and its analogue LDN-193189, the first class of BMP inhibitors, have CLUE scores of -90.02 and -91.86, respectively, while calyculin A, which has been shown to hyperphosphorylate β-catenin, has the most negative CLUE score (-99.93) among all the pharmacological perturbagens (S3 Table) [27, 28].

We then identified 20 genes in the LM gene signature that are opposed by LM therapeutics (Fig 4B). In order to elucidate the molecular pathways involved in the drug-disease interaction, we performed pathway analysis, which revealed that AKT, FLT3, and mTOR are among the upstream regulators associated with these genes (Fig 4C).

## Discussion

In this study, we present for the first time a LM-specific gene signature that is detectable in peripheral blood. This 421-gene signature not only stratifies LM subjects from healthy individuals but also reveals the genetic diversity that exists within LM. Of note, subjects 9 and 10, who present with severe LMs of the head and neck clustered together. Other than this, however, we noted little correlation between clustering and the clinical characteristics of the patients, nor was there a significant difference between microcytic and macrocytic subtypes (Fig 2, Table 1). Interestingly, others have also observed a poor correlation between the genetics of LM lesions (i.e. specific *PIK3CA* mutation) and clinical presentation, suggesting that molecular heterogeneity may not correlate with clinical heterogeneity [10].

Pathway analysis of our LM gene signature confirms the role of the PI3K/AKT/mTOR pathway in LM pathogenesis. The PI3K/AKT/mTOR pathway modulates numerous cellular processes including cell growth, cell proliferation and angiogenesis, and activating mutations in this pathway have been detected in LM tissues [10, 11]. AKT activation has also been found to increase oxidative phosphorylation and oxygen consumption by improving coupling of glucose metabolism to oxidative phosphorylation [29]. The detection of PI3K/AKT activation in peripheral blood suggests either the presence of circulating LM cells or the systemic impact of LM beyond the lesion. Though researchers have detected *PIK3CA* mutations in peripheral blood of patients with PROS [30, 31], to the best of our knowledge, there is no literature to date demonstrating the presence *PIK3CA* mutations in peripheral blood in isolated LM. Consistent with this, we did not detect *PIK3CA* mutations in any of our blood samples. This data may support a systemic impact of LM beyond the endothelial cells. Further studies are needed to evaluate the implications of systemic PI3K/AKT activation in LM.

The PI3K/AKT and MEK/ERK axes represent the two major pathways through which vascular endothelial growth factor-C (VEGF-C)/VEGF receptor-3 (VEGFR-3) signaling induces lymphangiogenesis. Our data suggest that key regulators of both pathways are associated with the LM gene signature: PIK3CD, PIK3R1, and MEK. *PIK3CD* and *PIK3R1* encode the catalytic subunit and the regulatory subunit of PI3K, respectively. *PIK3CD* encodes p110δ, the catalytic subunit of PI3Kδ, which is an isoform of PI3Kα encoded by *PIK3CA*. *PIK3R1* encodes three isoforms of the regulatory subunit of PI3Ks and has been found to play an important role in embryonic lymphangiogenesis [32]. Activation of the PI3K/AKT/mTOR and MEK/ERK pathways is further supported by higher expression of phospho-4E-BP1 and phospho-ERK, which are downstream of mTOR and MEK, respectively, in LECs of LM patients. Consistent with this, researchers have recently reported combined activation of PI3K/AKT and MEK/ERK pathways in LECs collected from LM patients [11].

We have also identified novel pathway alterations in LM, including the BMP, Eph/ephrin and Wnt/β-catenin pathways. BMPs belong to the transforming growth factor β family and play central roles in vascular and skeletal development [33]. In particular, BMP9 is an important regulator of lymphatic network maturation and lymphatic valve formation [34]. It is also known to upregulate ephrin B2, whose interaction with its receptor EphB4 is involved in constructing a mature lymphatic network from the lymphatic capillary plexus [35]. Wnts are a family of highly conserved, secreted glycoproteins that play essential roles in developmental processes such as proliferation, migration, differentiation and apoptosis [36]. A recent study [37] reported that *Wnt5a*-null-mice has defective sprouting of dermal lymphatics, demonstrating the importance of *Wnt5a* in regulating lymphangiogenesis. In our immunochemistry experiments, we observed nuclear localization of β-catenin, which lends further credence to the involvement of the Wnt/β-catenin pathway in LM. These pathways offer novel potential molecular targets for LM therapy.

In addition to the canonical regulators in LM such as PIK3CD, PIK3R1, and MEK, two additional upstream regulators were found: MTA3 and SOX11. Metastasis tumor antigen 3 (MTA3) is a member of a cancer progression-related protein family and has been found to be associated with lymph node involvement and lymphovascular space invasion in uterine non-endometrial cancer [38]. SOX11 is a transcription factor that plays an important role in embryonic neurogenesis and is overexpressed in central nervous system malignancies and mantle cell lymphomas [39]. These findings demonstrate the ability of our LM gene signature to identify novel upstream regulators that may be involved in LM pathogenesis.

We also performed computational drug repositioning analysis on our LM gene signature. As expected, sirolimus has the most negative CLUE score (i.e. the most dissimilar gene signature) among the known LM therapeutics and is in the top 2% of predicted FDA-approved drugs in CLUE. Prednisolone [40], sunitinib [41], and propranolol [13] have also shown success in treating LM as either a monotherapy or part of a combination therapy, and their CLUE scores represent the top 3 to 28% of the predicted FDA-approved drugs. Interestingly, sildenafil has a gene signature that is neither similar or dissimilar to LM signature, suggesting that the drug may function via a different mechanism of action. This finding supports the hypothesis by others that sildenafil exerts its therapeutic effects by inhibiting phosphodiesterase-5 and thereby inducing smooth muscle relaxation and subsequent vasodilation [14]. Also, the highly negative CLUE score of vincristine suggests angiogenic similarities between vascular tumors and LMs. Pathway analysis of 20 genes opposed by these drugs identifies AKT, FLT3, and mTOR as the upstream regulators associated with these genes. These molecules are not only related to LM pathogenesis but also consistent with the reported mechanisms of action of the drugs. For example, FLT3 is the molecular target of sunitinib, and FLT3 activation is known to upregulate the PI3K/AKT pathway [42].

In addition to confirming the known LM therapeutics, our LM gene signature suggests the therapeutic potential of BMP and Wnt modulators: dorsomorphin, LDN-193189, and calyculin A. Though these drugs are not approved by the FDA for human use, these results support the involvement of the BMP and Wnt pathways in LM and demonstrate the potential of the LM gene signature in facilitating drug discovery for LM. Other classes of FDA-approved drugs that may have therapeutic potential based on CLUE score include JAK inhibitors, calcium channel blockers, and KATP activators (S2 Table). Further studies are needed to investigate their possible clinical benefits in the treatment of LM.

In summary, we have discovered a LM-specific gene signature in peripheral blood and identified novel pathway alterations in LM, including oxidative phosphorylation, MEK/ERK, BMP, Wnt/β-catenin and ephrin signaling pathways. This LM gene signature not only enables differentiation of LM subjects from healthy individuals but also sheds light on the molecular heterogeneity that exists within LMs. We also performed computational drug repositioning analysis and confirmed the therapeutic potential of sirolimus and several other medications, which further validates the utility of this gene signature in exploring other targeted therapeutics. Our findings illuminate the molecular pathways involved in LM pathogenesis and demonstrate the potential of LM gene signatures to aid in non-invasive diagnosis, pathway analysis and therapeutic development. Efforts are underway to investigate the therapeutic potential of our predicted drugs in preclinical and clinical studies.

## Supporting information

S1 Fig Downstream target molecules that are differentially expressed in the serum samples of LM patients. Upstream regulators: (A) SOX11, (B) Mek, (C) MTA3, (D) PIK3CD, and (E) PIK3R1. Red, upregulated; green, downregulated; orange arrow, activation; blue arrow, inhibition; yellow arrow, data inconsistent with predicted state of downstream molecule; grey arrow, effect not predicted. Figure adapted from Ingenuity Pathway Analysis (IPA^®^ version 01–12, QIAGEN Redwood City).
(TIF)

S2 Fig Immunohistochemistry of normal skin of a healthy individual. (A) anti-phospho-4E-BP1 and (B) anti-phospho-ERK antibodies. Magnification: 10X.
(TIF)

S1 Table 421 genes that are differentially expressed in blood samples of LM patients. (DOCX)

S2 Table Top 40 FDA-approved drugs with most negative CLUE scores. Known LM therapeutics are shown in bold.
(DOCX)

S3 Table Top 40 preclinical drugs with most negative CLUE scores. (DOCX)
