# Supplementary figures and images for: Alterations of the MEK/ERK, BMP, and Wnt/β-catenin pathways detected in the blood of individuals with lymphatic malformations
Source: PLoS One. 2019 Apr 4;14(4):e0213872. doi: 10.1371/journal.pone.0213872 (PMC6448917; doi:10.1371/journal.pone.0213872)

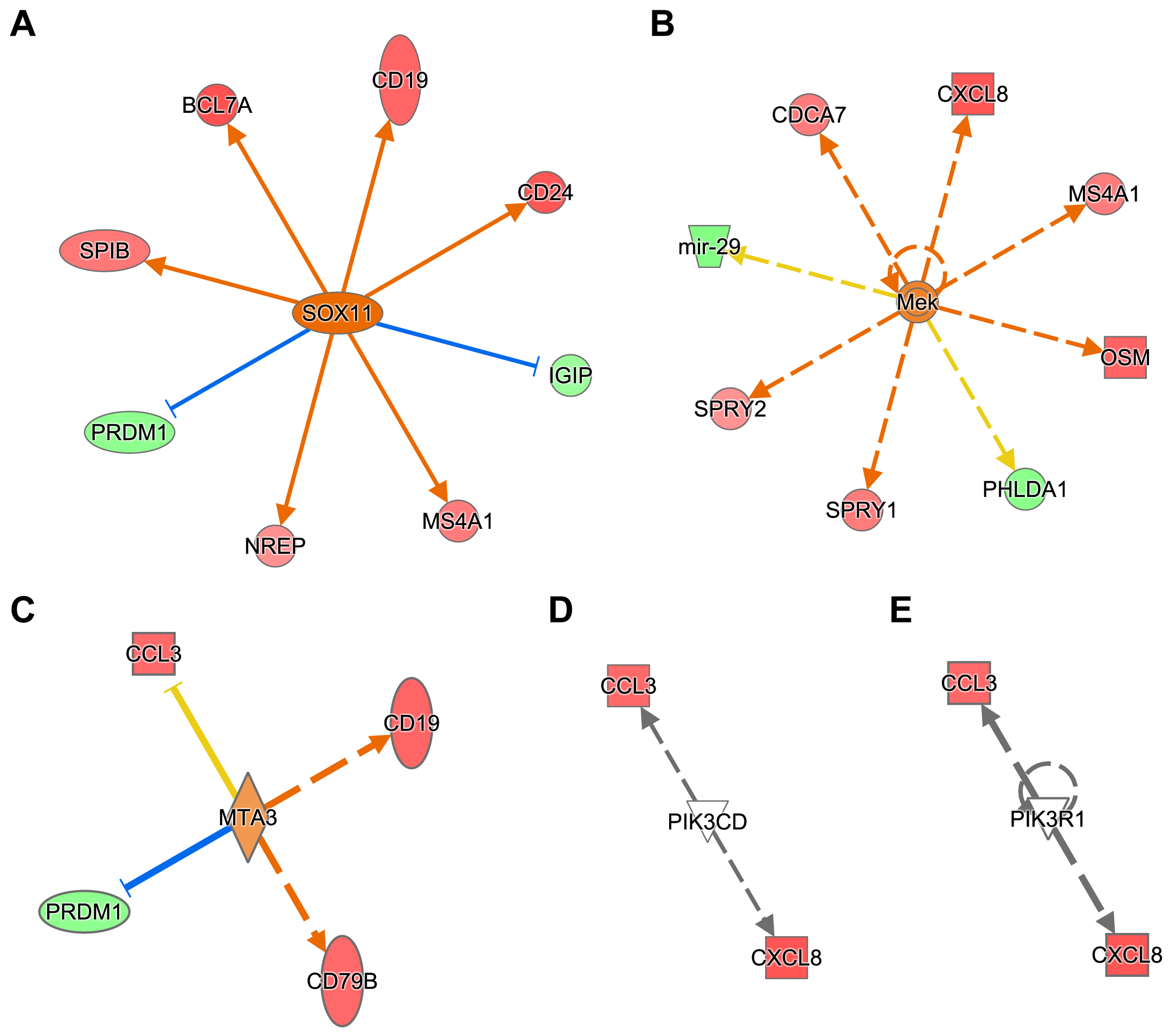

Supplement: S1 Fig — Upstream regulators: (A) SOX11, (B) Mek, (C) MTA3, (D) PIK3CD, and (E) PIK3R1. Red, upregulated; green, downregulated; orange arrow, activation; blue arrow, inhibition; yellow arrow, data inconsistent with predicted state of downstream molecule; grey arrow, effect not predicted. Figure adapted from Ingenuity Pathway Analysis (IPA® version 01–12, QIAGEN Redwood City). (TIF) [file pone.0213872.s001.tif]

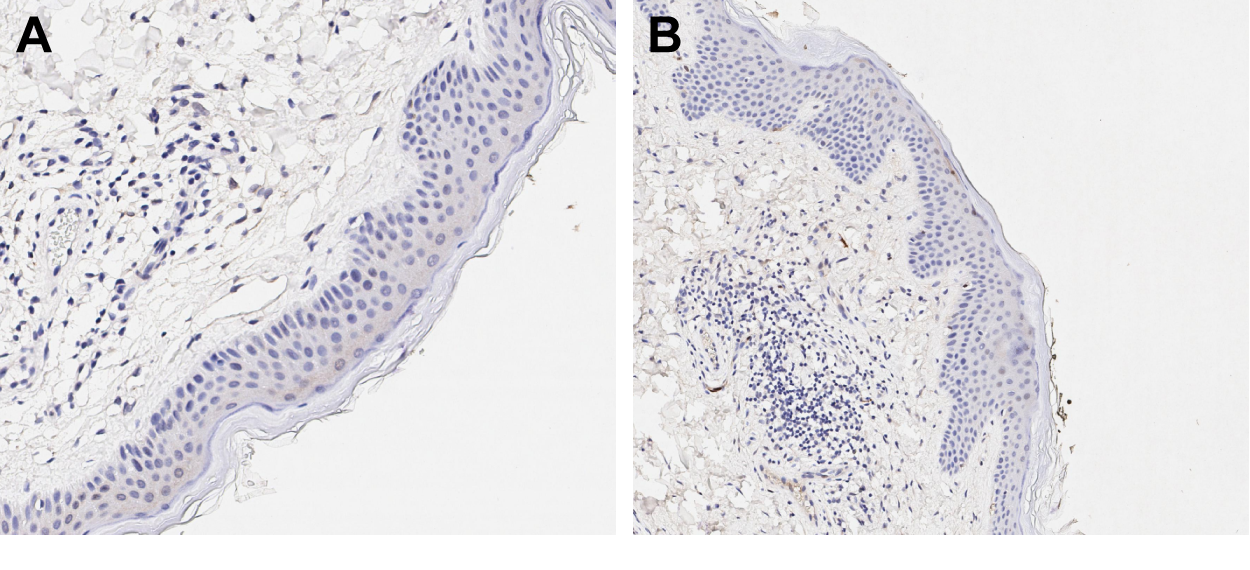

Supplement: S2 Fig — (A) anti-phospho-4E-BP1 and (B) anti-phospho-ERK antibodies. Magnification: 10X. (TIF) [file pone.0213872.s002.tif]
