# Supplementary material for: Alterations of the MEK/ERK, BMP, and Wnt/β-catenin pathways detected in the blood of individuals with lymphatic malformations
Source: PLoS One. 2019 Apr 4;14(4):e0213872. doi: 10.1371/journal.pone.0213872 (PMC6448917; doi:10.1371/journal.pone.0213872)
Supplement: S1 Table — (DOCX) [file pone.0213872.s003.docx]

**S1 Table. 421 Genes that are differentially expressed in blood samples of LM patients**

| **Gene** | **Log_2_FC** | **Adj. p-value^1^** |
| --- | --- | --- |
| FBLN2 | 1.324 | 2.55E-04 |
| CASC15 | 1.209 | 1.42E-03 |
| HIST1H3A | 1.16 | 3.93E-03 |
| ANOS1 | 1.137 | 5.46E-03 |
| PRKY | 1.101 | 2.46E-03 |
| MYZAP | 1.067 | 1.01E-02 |
| CR2 | 1.032 | 3.16E-03 |
| MIR650 | 1.027 | 1.57E-02 |
| LINC00167 | 0.996 | 1.87E-02 |
| TM4SF1 | 0.995 | 1.76E-02 |
| BMP3 | 0.992 | 1.91E-02 |
| BCL7A | 0.987 | 1.05E-02 |
| RASSF8 | 0.971 | 1.76E-02 |
| SNORA5A | 0.97 | 1.88E-02 |
| BRE-AS1 | 0.968 | 2.18E-02 |
| LOC221946 | 0.965 | 1.87E-02 |
| HIST1H2AK | 0.962 | 2.49E-02 |
| CXCL8 | 0.962 | 1.87E-02 |
| CD24 | 0.956 | 6.45E-03 |
| HIST1H2BN | 0.952 | 1.41E-02 |
| CHI3L2 | 0.952 | 1.44E-02 |
| SNORA45A | 0.947 | 2.87E-02 |
| POMC | 0.946 | 2.03E-02 |
| SNORA64 | 0.946 | 1.78E-02 |
| LINC01013 | 0.94 | 2.88E-02 |
| SMARCA5-AS1 | 0.94 | 2.87E-02 |
| LOC100289495 | 0.935 | 2.88E-02 |
| CDCA7L | 0.935 | 4.53E-03 |
| LOC100289511 | 0.931 | 2.91E-02 |
| HIST1H2BG | 0.919 | 1.87E-02 |
| RPPH1 | 0.915 | 2.83E-02 |
| HIST1H2AL | 0.91 | 2.90E-02 |
| DUSP2 | 0.909 | 3.39E-02 |
| TMEM107 | 0.899 | 2.07E-02 |
| LOC101929372 | 0.895 | 3.38E-02 |
| IFI44L | 0.894 | 3.61E-02 |
| HSPA2 | 0.893 | 3.71E-02 |
| **Gene** | **Log_2_FC** | **Adj. p-value^1^** |
| SNORA38 | 0.89 | 3.26E-02 |
| OSM | 0.884 | 3.92E-02 |
| PHGDH | 0.882 | 7.28E-03 |
| STOX1 | 0.88 | 3.92E-02 |
| SNORA80B | 0.879 | 3.92E-02 |
| RPL13AP20 | 0.875 | 2.84E-02 |
| CD19 | 0.873 | 2.56E-02 |
| HIST1H2BB | 0.87 | 3.94E-02 |
| SNORA74B | 0.864 | 3.27E-02 |
| TCL1A | 0.854 | 3.38E-02 |
| PLK2 | 0.852 | 4.32E-02 |
| USP18 | 0.851 | 3.39E-02 |
| MMP17 | 0.85 | 3.36E-02 |
| HIST1H1E | 0.85 | 3.90E-02 |
| CD79B | 0.849 | 2.59E-02 |
| CCL3 | 0.848 | 3.61E-02 |
| HIST2H2AB | 0.847 | 3.38E-02 |
| SSTR5-AS1 | 0.846 | 4.72E-02 |
| RNU11 | 0.845 | 2.55E-02 |
| SNORD17 | 0.843 | 3.94E-02 |
| HIST1H3D | 0.842 | 1.98E-02 |
| SNORA52 | 0.839 | 4.37E-02 |
| C12orf57 | 0.838 | 4.60E-02 |
| TACSTD2 | 0.833 | 4.96E-02 |
| HIST1H2AE | 0.824 | 3.53E-02 |
| HIST1H1B | 0.819 | 4.42E-02 |
| SCARNA6 | 0.817 | 4.94E-02 |
| SNORA74A | 0.817 | 2.86E-02 |
| HYAL3 | 0.815 | 3.38E-02 |
| PCDH9 | 0.815 | 2.96E-02 |
| SCARNA10 | 0.814 | 4.43E-02 |
| ZNF135 | 0.814 | 3.33E-02 |
| SOX4 | 0.809 | 3.52E-02 |
| HIST1H2BL | 0.809 | 4.62E-02 |
| PODXL2 | 0.806 | 3.09E-02 |
| HIST1H2BF | 0.804 | 1.87E-02 |
| SCARNA5 | 0.803 | 3.38E-02 |
| **Gene** | **Log_2_FC** | **Adj. p-value^1^** |
| SNORA5C | 0.798 | 4.43E-02 |
| EFNA1 | 0.797 | 4.49E-02 |
| HIST1H2AM | 0.797 | 4.37E-02 |
| COL24A1 | 0.793 | 4.37E-02 |
| RNVU1-19 | 0.792 | 3.38E-02 |
| SNORA23 | 0.788 | 3.92E-02 |
| BEND5 | 0.787 | 4.31E-02 |
| ZNF112 | 0.781 | 4.37E-02 |
| SOX5 | 0.781 | 4.67E-02 |
| IGLL5 | 0.78 | 4.37E-02 |
| P2RX5 | 0.777 | 4.14E-02 |
| CCDC157 | 0.775 | 1.87E-02 |
| AGRN | 0.774 | 1.58E-02 |
| PTCH2 | 0.773 | 4.32E-02 |
| PTK7 | 0.77 | 1.81E-02 |
| SNX22 | 0.769 | 3.16E-03 |
| SPIB | 0.766 | 3.38E-02 |
| HIST1H2BD | 0.762 | 3.87E-02 |
| ZNF418 | 0.76 | 2.16E-02 |
| CXXC5 | 0.754 | 3.94E-02 |
| CDCA7 | 0.752 | 2.49E-02 |
| RNVU1-20 | 0.746 | 4.45E-02 |
| MS4A1 | 0.745 | 4.94E-02 |
| SPRY1 | 0.74 | 3.38E-02 |
| RPS11 | 0.731 | 5.57E-03 |
| HIST2H2AC | 0.727 | 4.82E-02 |
| CCR9 | 0.725 | 1.77E-02 |
| PKIG | 0.724 | 4.43E-02 |
| FAM109B | 0.717 | 4.22E-02 |
| ADGRA3 | 0.716 | 3.90E-02 |
| CYB5A | 0.715 | 3.94E-02 |
| C9orf16 | 0.71 | 1.05E-02 |
| ABCB4 | 0.703 | 3.75E-02 |
| GAMT | 0.694 | 3.90E-02 |
| RPS2P32 | 0.687 | 4.49E-02 |
| HIST1H3I | 0.684 | 3.90E-02 |
| GGH | 0.681 | 3.92E-02 |
| FAM129C | 0.68 | 4.42E-02 |
| RPS15 | 0.667 | 2.41E-02 |
| **Gene** | **Log_2_FC** | **Adj. p-value^1^** |
| WDR34 | 0.66 | 2.96E-02 |
| LINC00865 | 0.659 | 3.39E-02 |
| MZT2B | 0.655 | 3.46E-02 |
| MIR181A1HG | 0.652 | 3.39E-02 |
| PLD4 | 0.651 | 1.68E-02 |
| UBE2S | 0.65 | 4.94E-02 |
| PTRHD1 | 0.649 | 1.87E-02 |
| MRPL55 | 0.635 | 1.56E-02 |
| TLE1 | 0.635 | 4.49E-02 |
| SERPINF1 | 0.635 | 4.31E-02 |
| NREP | 0.621 | 3.15E-03 |
| SPRY2 | 0.613 | 3.08E-02 |
| ACOT7 | 0.61 | 4.67E-02 |
| SUSD3 | 0.606 | 2.90E-02 |
| CD14 | 0.604 | 1.87E-02 |
| HIST1H2BK | 0.591 | 4.31E-02 |
| SPNS1 | 0.588 | 4.32E-02 |
| MCM2 | 0.583 | 4.49E-02 |
| RFC4 | 0.581 | 3.92E-02 |
| STK16 | 0.57 | 1.96E-03 |
| BOP1 | 0.568 | 3.94E-02 |
| NTHL1 | 0.564 | 4.43E-02 |
| EXOSC4 | 0.562 | 3.38E-02 |
| DUS2 | 0.556 | 5.73E-03 |
| MAP10 | 0.556 | 4.42E-02 |
| ATP5C1 | 0.553 | 3.27E-02 |
| PC | 0.553 | 3.92E-02 |
| SNX25 | 0.549 | 4.94E-02 |
| TM7SF2 | 0.549 | 6.39E-03 |
| RAD54B | 0.545 | 4.67E-02 |
| UQCRH | 0.542 | 2.55E-02 |
| PTPMT1 | 0.542 | 3.94E-02 |
| DHX58 | 0.54 | 3.39E-02 |
| PMF1 | 0.538 | 3.52E-03 |
| NHLRC4 | 0.537 | 4.67E-02 |
| STRBP | 0.534 | 1.87E-02 |
| PMS2P1 | 0.533 | 4.68E-02 |
| SPINT2 | 0.52 | 1.33E-02 |
| ZDHHC14 | 0.518 | 4.79E-02 |
| **Gene** | **Log_2_FC** | **Adj. p-value^1^** |
| DEAF1 | 0.517 | 4.38E-02 |
| UROD | 0.517 | 3.75E-02 |
| C8orf82 | 0.517 | 3.90E-02 |
| CHEK1 | 0.515 | 3.39E-02 |
| NDUFA2 | 0.514 | 3.92E-02 |
| ZNF511 | 0.508 | 3.38E-02 |
| COMT | 0.505 | 3.11E-02 |
| CAPG | 0.505 | 1.03E-02 |
| ARMC6 | 0.502 | 2.87E-02 |
| CCDC94 | 0.501 | 3.82E-02 |
| MRPL37 | 0.499 | 2.87E-02 |
| CCS | 0.497 | 2.63E-03 |
| MUTYH | 0.496 | 3.87E-02 |
| SLC50A1 | 0.493 | 4.94E-02 |
| PCCB | 0.492 | 9.07E-03 |
| IKZF2 | 0.489 | 4.78E-02 |
| NUCB2 | 0.486 | 1.52E-02 |
| WDR83OS | 0.486 | 3.90E-02 |
| MRPL1 | 0.478 | 4.99E-02 |
| CDK5 | 0.47 | 2.90E-02 |
| PIN1 | 0.463 | 3.94E-02 |
| POLD1 | 0.463 | 3.75E-02 |
| ZDHHC4 | 0.462 | 4.94E-02 |
| RFXANK | 0.461 | 1.76E-02 |
| ATF4 | 0.46 | 3.41E-02 |
| EIF2D | 0.46 | 3.83E-02 |
| BCKDHA | 0.459 | 1.17E-02 |
| BAD | 0.456 | 3.92E-02 |
| CHST2 | 0.455 | 3.38E-02 |
| NR1H2 | 0.453 | 3.08E-02 |
| PPP1R35 | 0.451 | 4.94E-02 |
| NDUFS7 | 0.45 | 4.94E-02 |
| VPS51 | 0.449 | 3.75E-02 |
| HMG20B | 0.449 | 2.88E-02 |
| GAPDH | 0.449 | 3.09E-02 |
| ARMCX6 | 0.448 | 4.60E-02 |
| PI4K2A | 0.44 | 3.38E-02 |
| CHCHD3 | 0.438 | 1.87E-02 |
| STX18 | 0.437 | 4.31E-02 |
| **Gene** | **Log_2_FC** | **Adj. p-value^1^** |
| ZNF768 | 0.433 | 1.87E-02 |
| MIF4GD | 0.432 | 4.43E-02 |
| B4GALT7 | 0.428 | 4.31E-02 |
| WDSUB1 | 0.426 | 4.53E-02 |
| TSSC4 | 0.425 | 2.91E-02 |
| GPAA1 | 0.419 | 8.82E-04 |
| PSMB7 | 0.412 | 4.75E-02 |
| SCAMP3 | 0.41 | 4.32E-02 |
| SSNA1 | 0.409 | 4.43E-02 |
| UQCRC1 | 0.408 | 4.94E-02 |
| CCND3 | 0.408 | 1.50E-02 |
| DDX49 | 0.406 | 2.13E-02 |
| SCPEP1 | 0.405 | 4.43E-02 |
| ENO1 | 0.4 | 1.68E-02 |
| PEX16 | 0.393 | 4.28E-02 |
| RPS19BP1 | 0.386 | 3.92E-02 |
| FBXW5 | 0.384 | 9.07E-03 |
| ADRM1 | 0.381 | 3.41E-02 |
| MCM7 | 0.381 | 9.41E-03 |
| NDUFA10 | 0.38 | 3.38E-02 |
| RNF181 | 0.375 | 4.88E-02 |
| RMND5B | 0.373 | 2.26E-02 |
| RPS6KB2 | 0.366 | 6.39E-03 |
| CPSF3 | 0.361 | 2.57E-02 |
| PARP10 | 0.354 | 4.43E-02 |
| PSMC4 | 0.353 | 3.33E-02 |
| LRRC8D | 0.349 | 3.75E-02 |
| TXNDC11 | 0.346 | 3.71E-02 |
| COG4 | 0.345 | 1.16E-02 |
| LRRC59 | 0.344 | 3.94E-02 |
| PSMD3 | 0.341 | 4.68E-02 |
| TBC1D9B | 0.334 | 1.49E-02 |
| CHD1L | 0.334 | 3.94E-02 |
| FAM134C | 0.334 | 3.27E-02 |
| CCDC53 | 0.33 | 4.46E-02 |
| BAP1 | 0.329 | 3.16E-03 |
| ABCB7 | 0.326 | 2.84E-02 |
| ANKRA2 | 0.322 | 3.83E-02 |
| NUDT22 | 0.321 | 3.33E-02 |
| **Gene** | **Log_2_FC** | **Adj. p-value^1^** |
| DGKD | 0.32 | 4.62E-02 |
| INPP5B | 0.32 | 3.16E-03 |
| CD99L2 | 0.319 | 3.90E-02 |
| MUS81 | 0.318 | 4.37E-02 |
| P3H1 | 0.317 | 1.91E-02 |
| MAPRE2 | 0.314 | 4.94E-02 |
| GTF3C5 | 0.31 | 4.46E-02 |
| ANAPC5 | 0.309 | 9.10E-03 |
| LIMD2 | 0.308 | 3.92E-02 |
| NUP88 | 0.306 | 4.43E-02 |
| WRNIP1 | 0.301 | 4.62E-02 |
| KAT8 | 0.295 | 2.49E-02 |
| NMT1 | 0.292 | 3.53E-02 |
| EFTUD2 | 0.27 | 3.94E-02 |
| MFF | 0.27 | 4.43E-02 |
| CORO1A | 0.268 | 2.26E-02 |
| ORC3 | 0.262 | 4.82E-02 |
| PPP6R2 | 0.257 | 3.92E-02 |
| APEH | 0.255 | 3.99E-02 |
| TCF25 | 0.244 | 1.52E-02 |
| VPS18 | 0.235 | 3.38E-02 |
| CDC37 | 0.23 | 4.31E-02 |
| OSBPL9 | 0.184 | 3.54E-02 |
| KHDRBS1 | -0.198 | 3.94E-02 |
| YTHDF3 | -0.222 | 2.88E-02 |
| THAP6 | -0.27 | 3.38E-02 |
| ELMOD2 | -0.271 | 3.27E-02 |
| ZNF611 | -0.272 | 3.38E-02 |
| KLHL28 | -0.273 | 8.67E-03 |
| GTF2A1 | -0.276 | 2.77E-02 |
| MPHOSPH8 | -0.28 | 6.39E-03 |
| APH1B | -0.284 | 4.94E-02 |
| NFYA | -0.285 | 4.19E-02 |
| GXYLT1 | -0.287 | 4.43E-02 |
| C18orf25 | -0.299 | 2.64E-02 |
| FMR1 | -0.305 | 3.38E-02 |
| SEC24D | -0.307 | 4.94E-02 |
| ATL3 | -0.31 | 9.10E-03 |
| PSMD12 | -0.31 | 1.17E-02 |
| **Gene** | **Log_2_FC** | **Adj. p-value^1^** |
| TAF1D | -0.316 | 1.75E-02 |
| XIAP | -0.317 | 3.38E-02 |
| ITGAL | -0.332 | 3.47E-02 |
| CREB1 | -0.332 | 4.94E-02 |
| FEM1C | -0.334 | 3.33E-02 |
| SLC35F5 | -0.336 | 3.38E-02 |
| GFPT1 | -0.338 | 2.92E-02 |
| RBMXL1 | -0.338 | 3.39E-02 |
| SCRN3 | -0.35 | 1.03E-02 |
| MDM4 | -0.351 | 3.52E-02 |
| LYSMD3 | -0.352 | 2.88E-02 |
| SIN3A | -0.355 | 4.44E-02 |
| RBM12B | -0.363 | 1.75E-02 |
| SRGAP2B | -0.369 | 1.98E-02 |
| CALML4 | -0.37 | 3.75E-02 |
| HNRNPLL | -0.371 | 4.66E-02 |
| PRKAG2 | -0.373 | 1.06E-02 |
| NEDD9 | -0.381 | 4.37E-02 |
| MAPK1IP1L | -0.384 | 2.87E-02 |
| FAM8A1 | -0.388 | 4.78E-02 |
| BTBD2 | -0.39 | 4.44E-02 |
| TAPBP | -0.399 | 3.94E-02 |
| ABCA5 | -0.408 | 4.90E-02 |
| KCNA3 | -0.408 | 2.59E-02 |
| ZFP36L2 | -0.416 | 3.38E-02 |
| WDFY2 | -0.416 | 3.38E-02 |
| UST | -0.419 | 4.82E-02 |
| RBM12B-AS1 | -0.428 | 3.90E-02 |
| PRRC2C | -0.43 | 3.88E-02 |
| PPTC7 | -0.43 | 2.03E-02 |
| LXN | -0.433 | 3.94E-02 |
| PLAGL2 | -0.437 | 4.94E-02 |
| LINC00893 | -0.437 | 4.43E-02 |
| ZFX | -0.439 | 1.87E-02 |
| RAB14 | -0.441 | 2.87E-02 |
| EIF5B | -0.445 | 2.57E-02 |
| EIF1AX | -0.448 | 1.03E-02 |
| MALAT1 | -0.45 | 3.75E-02 |
| STS | -0.451 | 3.27E-02 |
| **Gene** | **Log_2_FC** | **Adj. p-value^1^** |
| PRKX | -0.452 | 1.87E-02 |
| ELMSAN1 | -0.454 | 3.38E-02 |
| PRDM8 | -0.458 | 4.68E-02 |
| SPRED1 | -0.459 | 1.86E-02 |
| SH3BP1 | -0.463 | 2.83E-02 |
| ZRSR2 | -0.474 | 4.32E-03 |
| CARD8 | -0.475 | 3.38E-02 |
| CCDC30 | -0.481 | 3.39E-02 |
| HERC3 | -0.486 | 4.43E-02 |
| C14orf159 | -0.487 | 1.81E-02 |
| FYB | -0.494 | 3.38E-02 |
| ZNF652 | -0.503 | 4.95E-02 |
| LPCAT4 | -0.504 | 4.35E-02 |
| PCNXL2 | -0.51 | 2.35E-02 |
| ANKHD1 | -0.513 | 3.10E-02 |
| NAV1 | -0.52 | 2.93E-02 |
| WIPF1 | -0.523 | 3.94E-02 |
| CA5B | -0.524 | 9.69E-05 |
| CCDC65 | -0.537 | 2.88E-02 |
| GPRIN3 | -0.54 | 3.10E-03 |
| CBLL1 | -0.541 | 7.59E-05 |
| RNF185 | -0.544 | 1.96E-03 |
| RUNX2 | -0.544 | 3.92E-02 |
| APOL1 | -0.547 | 4.32E-02 |
| RORA | -0.552 | 1.29E-02 |
| ENO2 | -0.552 | 6.62E-03 |
| MAF | -0.554 | 7.59E-05 |
| PDIA3P1 | -0.556 | 3.90E-02 |
| LOC100506606 | -0.562 | 4.94E-02 |
| MAGI3 | -0.564 | 3.94E-02 |
| EOGT | -0.565 | 4.53E-04 |
| HNRNPH3 | -0.569 | 7.59E-05 |
| FAM92A1 | -0.574 | 3.38E-02 |
| APOBR | -0.574 | 4.43E-02 |
| TCF7L2 | -0.579 | 2.35E-02 |
| LOC148696 | -0.582 | 3.26E-02 |
| ANKRD36BP1 | -0.585 | 1.50E-02 |
| IGIP | -0.586 | 3.94E-02 |
| SPDYE1 | -0.589 | 4.37E-02 |
| **Gene** | **Log_2_FC** | **Adj. p-value^1^** |
| CD84 | -0.592 | 7.08E-03 |
| RNF157 | -0.597 | 4.59E-02 |
| GNAL | -0.598 | 2.88E-02 |
| FAS-AS1 | -0.599 | 3.83E-02 |
| DUSP16 | -0.602 | 1.70E-03 |
| IL2RA | -0.604 | 2.87E-02 |
| AMDHD1 | -0.613 | 3.94E-02 |
| PRRG4 | -0.618 | 3.99E-02 |
| SEC61A2 | -0.619 | 3.87E-02 |
| EMBP1 | -0.62 | 3.38E-02 |
| MYADM | -0.624 | 3.75E-02 |
| CXorf57 | -0.633 | 4.94E-02 |
| TRIM2 | -0.644 | 2.03E-02 |
| CYB561 | -0.655 | 1.03E-02 |
| PRDM1 | -0.658 | 3.53E-02 |
| LINC00899 | -0.66 | 3.26E-02 |
| TRPC1 | -0.677 | 2.57E-02 |
| HCG26 | -0.68 | 3.27E-02 |
| ZSWIM5 | -0.68 | 1.50E-02 |
| PVRL3 | -0.685 | 4.99E-02 |
| PSPH | -0.686 | 3.38E-02 |
| LOC100130992 | -0.69 | 2.14E-03 |
| ERV3-1 | -0.691 | 9.03E-03 |
| AGAP7P | -0.691 | 4.68E-02 |
| PRR34 | -0.691 | 4.59E-02 |
| PHLDA1 | -0.692 | 1.76E-02 |
| NLGN3 | -0.693 | 4.49E-02 |
| TNFRSF4 | -0.696 | 3.30E-02 |
| TJP3 | -0.696 | 2.35E-02 |
| ASPRV1 | -0.707 | 3.87E-02 |
| SDHAP2 | -0.716 | 2.40E-03 |
| RRS1-AS1 | -0.72 | 1.87E-02 |
| SPACA6P | -0.726 | 2.07E-02 |
| MIR29C | -0.727 | 1.91E-02 |
| PIWIL2 | -0.728 | 4.43E-02 |
| EPHB1 | -0.73 | 3.33E-02 |
| KIF5C | -0.732 | 4.82E-02 |
| ZNF483 | -0.741 | 2.14E-03 |
| SLC12A1 | -0.742 | 4.51E-02 |
| **Gene** | **Log_2_FC** | **Adj. p-value^1^** |
| SYCP3 | -0.755 | 1.75E-02 |
| WNT4 | -0.763 | 2.96E-02 |
| ZNF117 | -0.766 | 2.31E-03 |
| LOC101929526 | -0.767 | 3.90E-02 |
| CHN1 | -0.77 | 4.37E-02 |
| MIR4257 | -0.782 | 3.72E-02 |
| PTPN13 | -0.786 | 1.27E-02 |
| TPPP | -0.797 | 1.87E-02 |
| ADAMTS1 | -0.799 | 3.38E-02 |
| LOC100132111 | -0.806 | 1.29E-02 |
| LINC00943 | -0.811 | 4.56E-02 |
| EDARADD | -0.813 | 3.39E-02 |
| SCRT2 | -0.815 | 4.81E-02 |
| ANKUB1 | -0.821 | 3.52E-02 |
| CASP1P2 | -0.855 | 3.08E-02 |
| C14orf132 | -0.885 | 3.52E-02 |
| SAMD12 | -0.895 | 1.74E-02 |
| CFH | -0.932 | 1.29E-02 |
|  |  |  |
| **Gene** | **Log_2_FC** | **Adj. p-value^1^** |
| MID2 | -0.96 | 8.59E-03 |
| MCOLN3 | -0.97 | 1.87E-02 |
| NAP1L2 | -0.972 | 6.62E-03 |
| NEO1 | -0.979 | 7.33E-04 |
| ACE | -0.997 | 8.92E-03 |
| LOC653513 | -1.036 | 2.63E-03 |
| CCR8 | -1.051 | 6.76E-03 |
| SOGA3 | -1.093 | 1.34E-04 |
| ZNF208 | -1.099 | 4.53E-04 |
| FAAHP1 | -1.103 | 3.16E-03 |
| FAM19A1 | -1.175 | 1.82E-04 |
| CACNA1I | -1.185 | 1.86E-05 |
| CLIC5 | -1.208 | 1.86E-05 |
| ACVR1C | -1.23 | 4.53E-04 |
| LINC00824 | -1.32 | 7.59E-05 |
| PPP4R4 | -1.329 | 4.53E-04 |
| NEFL | -1.568 | 9.58E-06 |
| Abbreviations: FC, fold change.  ^1^Benjamini-Hochberg adjusted p-value. | | |
