# Supplementary material for: Alterations of the MEK/ERK, BMP, and Wnt/β-catenin pathways detected in the blood of individuals with lymphatic malformations
Source: PLoS One. 2019 Apr 4;14(4):e0213872. doi: 10.1371/journal.pone.0213872 (PMC6448917; doi:10.1371/journal.pone.0213872)
Supplement: S2 Table — Known LM therapeutics are shown in bold. (DOCX) [file pone.0213872.s004.docx]

**S2 Table. Top 40 FDA-approved drugs with most negative CLUE scores.** Known LM therapeutics are shown in bold.

| **CLUE Score** | **Name** | **Description** |
| --- | --- | --- |
| -96.58 | Bisacodyl | Laxative |
| -94.64 | Piperacillin | Bacterial cell wall synthesis inhibitor |
| -93.99 | Rimantadine | Antiviral |
| -91.69 | Cefotaxime | Bacterial cell wall synthesis inhibitor |
| -90.69 | Ruxolitinib | JAK inhibitor |
| -89.81 | Nitrazepam | Benzodiazepine receptor agonist |
| -88.2 | Doxorubicin | Topoisomerase inhibitor |
| -87.42 | Nicardipine | Calcium channel blocker |
| -86.65 | Digoxin | ATPase inhibitor |
| -86.58 | Digitoxin | ATPase inhibitor |
| -84.96 | Mafenide | Carbonic anhydrase inhibitor |
| -84.67 | Moclobemide | Monoamine oxidase inhibitor |
| **-84.66** | **Sirolimus** | **mTOR inhibitor** |
| -83.83 | Niclosamide | DNA replication inhibitor |
| -83.79 | Vecuronium | Acetylcholine receptor antagonist |
| -83.13 | Clopidogrel | Purinergic receptor antagonist |
| -82.70 | Vincristine | Tubulin inhibitor |
| -81.69 | Etodolac | Cyclooxygenase inhibitor |
| -81.5 | Formestane | Aromatase inhibitor |
| -81.33 | Minoxidil | KATP activator |
| -80.62 | Nalbuphine | Opioid receptor agonist |
| -79.30 | Exemestane | Aromatase inhibitor |
| -78.97 | Diflunisal | Prostanoid receptor antagonist |
| -78.55 | Pitavastatin | HMGCR inhibitor |
| -78.4 | Parachlorophenol | Anti-infective |
| **-78.24** | **Prednisolone** | **Glucocorticoid receptor agonist** |
| -77.54 | Ethinylestradiol | Estrogen receptor agonist |
| -76.51 | Clarithromycin | Bacterial 50S ribosomal subunit inhibitor |
| -76.44 | Thiothixene | Dopamine receptor antagonist |
| -75.08 | Sulfacetamide | PABA antagonist |
| -74.62 | Hydrocortisone | Glucocorticoid receptor agonist |
| -74.2 | Fluvoxamine | Selective serotonin reuptake inhibitor (SSRI) |
| -73.74 | Amodiaquine | Histamine receptor agonist |
| -73.04 | Amlodipine | Calcium channel blocker |
| -72.48 | Crizotinib | ALK inhibitor |
| -71.12 | Yohimbine | Adrenergic receptor antagonist |
| -71.11 | Balsalazide | Cyclooxygenase inhibitor |
| -70.89 | Everolimus | mTOR inhibitor |
| -70.53 | Ziprasidone | Dopamine receptor antagonist |
| -70.01 | Floxuridine | DNA synthesis inhibitor |
| Following incidences have been removed due to duplication: vincristine (-77.75), hydrocortisone (-71.51). | | |
