# Supplementary material for: Alterations of the MEK/ERK, BMP, and Wnt/β-catenin pathways detected in the blood of individuals with lymphatic malformations
Source: PLoS One. 2019 Apr 4;14(4):e0213872. doi: 10.1371/journal.pone.0213872 (PMC6448917; doi:10.1371/journal.pone.0213872)
Supplement: S3 Table — (DOCX) [file pone.0213872.s005.docx]

**S3 Table. Top 40 preclinical drugs with most negative CLUE scores.**

| **CLUE Score** | **Name** | **Description** |
| --- | --- | --- |
| -99.93 | calyculin | Protein phosphatase inhibitor |
| -97.79 | genipin | Choleretic agent |
| -95.35 | BX-912 | Pyruvate dehydrogenase kinase inhibitor |
| -95.24 | linifanib | PDGFR receptor inhibitor |
| -94.98 | helveticoside | ATPase inhibitor |
| -94.52 | androsta-1,4-dien-3,17-dione | Aromatase inhibitor |
| -94.25 | forskolin | Adenylyl cyclase activator |
| -93.16 | digitoxigenin | ATPase inhibitor |
| -93.12 | strophanthidin | ATPase inhibitor |
| -92.85 | ellipticine | Topoisomerase inhibitor |
| -92.6 | manumycin-a | Farnesyltransferase inhibitor |
| -91.97 | ochratoxin-a | Phenylalanyl tRNA synthetase inhibitor |
| -91.86 | LDN-193189 | Serine/threonine kinase inhibitor |
| -91.1 | geldanamycin | HSP inhibitor |
| -90.37 | PI-103 | MTOR inhibitor |
| -90.08 | bucladesine | Adenosine receptor agonist |
| -90.02 | dorsomorphin | AMPK inhibitor |
| -89.97 | benperidol | Dopamine receptor antagonist |
| -89.83 | NSC-3852 | HDAC inhibitor |
| -89.71 | puromycin | Protein synthesis inhibitor |
| -89.57 | RO-90-7501 | Beta amyloid inhibitor |
| -88.44 | z-leu3-VS | Proteasome inhibitor |
| -88.27 | FTI-276 | Farnesyltransferase inhibitor |
| -87.98 | LE-300 | Dopamine receptor antagonist |
| -87.77 | andarine | Androgen receptor modulator |
| -87.5 | narciclasine | Coflilin signaling pathway activator |
| -87.48 | NVP-AUY922 | HSP inhibitor |
| -87.32 | FCCP | Mitochondrial OxPhos uncoupler |
| -87.27 | thiostrepton | FOXM1 inhibitor |
| -87.17 | VU-0418946-1 | HIF modulator |
| -86.83 | BNTX | Opioid receptor antagonist |
| -86.74 | isoflupredone | Glucocorticoid receptor agonist |
| -86.65 | KU-C103885 | inhibitor |
| -86.52 | radicicol | HSP inhibitor |
| -86.36 | BRD-K00313977 | - |
| -86.22 | PAC-1 | Caspase activator |
| -85.93 | kinetin-riboside | Apoptosis stimulant |
| -85.68 | NSC-632839 | Ubiquitin specific protease inhibitor |
| -85.58 | H-9 | PKA inhibitor |
| -85.55 | parbendazole | Tubulin inhibitor |
| Abbreviations: OxPhos, oxidative phosphorylation; CFTR, cystic fibrosis transmembrane conductance regulator | | |
